# ACL reconstruction with unicondylar replacement in knee with functional instability and osteoarthritis

**DOI:** 10.1186/1749-799X-4-43

**Published:** 2009-12-17

**Authors:** Srikrishna RSR Krishnan, Ray Randle

**Affiliations:** 1 Department of Orthopaedics, John Flynn Medical center, Inland Drive, Tugun, Qld - 4224, Australia

## Abstract

Severe symptomatic osteoarthritis in young and active patients with pre-existing deficiency of the anterior cruciate ligament and severe functionally instability is a difficult subgroup to manage. There is considerable debate regarding management of young patients with isolated unicompartment osteoarthritis and concomitant ACL deficiency. A retrospective analysis of was done in 9 patients with symptomatic osteoarthritis with ACL deficiencies and functional instability that were treated with unicompartment knee arthroplasty and ACL reconstruction between April 2002 and June 2005. The average arc of flexion was 119° (range 85° to 135°) preoperatively and 125° (range 105° to 140°). There were no signs of instability during the follow up of patients. No patients in this group were reoperated. In this small series we have shown that instability can be corrected and pain relieved by this combined procedure.

## Background

Isolated unicompartmental osteoarthritis of the knee is common. Operative treatment varies from high tibial osteotomy, unicompartmental knee replacement and total knee replacement according to the age of the patient and the level of activity [1]. Severe osteoarthritis of one compartment in young and active patients with pre-existing deficiency of the anterior cruciate ligament (ACL) and severe functionally instability is a difficult subgroup to manage [2].

There is considerable debate regarding management of young patients with isolated unicompartment osteoarthritis and concomitant ACL deficiency. The aim of the treatment should be to offer a procedure that will give lasting relief of symptoms and will not compromise any future surgery. Various surgical options have been described, including arthroscopic debridement, reconstruction of the ACL, high tibial osteotomy with or without ACL reconstruction, unicompartmental knee arthroplasty and total knee replacement [2]. None of these address the two major symptoms apart from total knee replacement.

The advantages of unicompartmental arthroplasty over total knee replacement are preservation of bone stock, less invasive surgery, minimal blood loss, faster recovery, better range of movement and more physiological function [3]. It is also more cost-effective than total knee replacement [4].

Recent studies have shown that, with the proper patient selection and surgical technique, UKA can have performance and survivorship comparable with total knee arthroplasty or high tibial osteotomy [5].

The anterior cruciate ligament (ACL) is the primary restraint to anterior tibial translation in the native knee [6]. It has been suggested that the ACL also plays an important role in the successful outcome of UKA [7-9]. Unicompartmental knee arthroplasty can provide disappointing long-term results when the ACL is deficient [10,11].

Good fellow found a greater incidence of failure of mobile-bearing UKA when the ACL was deficient [7]. A nonfunctional ACL was assumed to cause abnormal kinematics of the knee after UKA [12].

It may be important to divide the ACL deficient group into two subgroups. Firstly those patients with a prior, traumatic ACL tear and functional instability and second those patients with attrition of their ACL, without a concomitant capsule tear and in many instances some arthritis associated capsule stiffness. These patients do not have functional instability related to their ACL deficiency. These two separate groups may explain why some series have found poor results with ACL deficiency and other no difference

The majority of failures were because of tibial loosening, which tended to occur early, with a 21% rate of revision observed by two years [7]. It was proposed that this loosening may have resulted from eccentric or increased loading caused by posterior femoral subluxation or instability [7]. It was reasoned that if the posterior subluxation and instability could be prevented by reconstruction of the ACL, it might reduce the incidence of tibial loosening in this setting.

In the recent series, Pandit confirmed that the normal kinematics is restored in the ACL Deficient arthritic knee by combing ACLR and Oxford UKA. It is probably because the kinematics is restored that the patients who have had an ACLR and UKA have been able to achieve such a high level of function.

We report the early term results of fixed bearing unicompartmental knee arthroplasty in patients with isolated one compartment osteoarthritis and concomitant ACL deficiency with functional instability, in whom ligament reconstruction was undertaken as a combined procedure.

## Methods

We carried out a retrospective analysis of 9 patients operated on by the senior author (RR) with severe symptomatic osteoarthritis, ACL deficient and functional instability that were treated with unicompartmental knee arthroplasty and ACL reconstruction between April 2002 and June 2005.

The inclusion criteria were a range of motion of at least 90° with a flexion contracture of <15°, minimal pain at rest, positive lachmann and pivot shift test and an age of more than fifty years. The exclusion criteria were inflammatory arthritis, hemochromatosis, chondrocalcinosis, hemophilia, patellofemoral joint symptoms, a positive patellar grind test.

No patient was lost to follow-up. The average duration of follow-up for these nine patients was two years (range one to five years).

All patients were independently assessed clinically using the Oxford Knee score [13], the Knee Society score [14], and the Womac scoring system [15].

Radiographic analysis included measurement of the mechanical axis, measurement of the femorotibial axis, and assessment of the degree of correction. The cement interfaces were evaluated for the presence and extent of radiolucent lines in each zone. The positions of interference screws were evaluated. Sequential radiographs were reviewed for evidence of component subsidence, change of position and the position of the interference screws.

### Surgical Procedure

A straight anterior skin incision and medial parapatellar capsular incision were used. Intravenous antibiotic prophylaxis and antibiotic- loaded acrylic cement (Palacos with gentamicin) were used. Extramedullary instruments were used to guide tibial resection.

Allegretto (Zimmer) fixed bearing prosthesis was used for 5 patients and Preservation fixed bearing (Depuy) for 4 patients. The femoral component of the unicondylar prosthesis is made of cobalt-chromium alloy. The tibial component was all-polyethylene (preservation) and metal backed (Allegretto).

The procedures were all performed with preparation of the femoral and tibial surfaces first in the usual manner for unicompartmental arthroplasty. The tibial and femoral tunnels for the ACL graft were drilled in the same manner and the same position as conventional arthroscopic ACL reconstruction using jigs and cannulated drills. The graft was passed with the trial implants in position and the isometricity checked. The graft was left in position and the definitive implants cemented. The graft was then tensioned and fixed with interference screws.

The operation was performed in this order to minimize graft damage during bone preparation and to enable correct graft tensioning with the joint space restored.

With regard to ACL placement there are 3 areas of potential impingement.

These are

1. Impingement on the tibial implant

2. Impingement on the lateral intercondylar notch wall

3. Impingement on the PCL.

Impingement on the tibial prosthesis is avoided by placing the implants before the tibial tunnel is drilled. A guide is used with a K wire positioned and the reamer run over the K wire. Medial-lateral placement proximally and distally is important, just as in standard ACL reconstruction, to avoid impingement on the notch wall or the PCL. The femoral tunnel is normally drilled at the 10 o'clock position and the tibial tunnel positioned off the PCL and enabling the graft to be equidistant to the notch wall and the PCL. This is more difficult where the notch is very narrow and may occasionally require notchplasty.

The medial third of patella tendon was chosen as the graft in most of the patients because it was able to be harvested through the operation incision reducing operative morbidity. The more traditional middle third of patella tendon was not used because of the risk of devascularisation of the medial third remaining. The one patient who had a hamstring graft was a carpet layer who used his knee to kick his carpet laying tool and it was felt that a patella procedure may have made that area more sensitive.

Drainage was removed within 24 hours. The patients were mobilized the first day after surgery by use of 2 crutches and supervised by a physiotherapist. No postoperative bracing was used. Patients were allowed full weight bearing on the operated leg from the first postoperative day. Hospital stay began 1 day before surgery and lasted a mean of 4 days. The Standard rehabilitation protocol for ACL reconstruction was followed.

## Results

There were 9 patients in this group. Seven patients had unicompartmental replacement with ACL reconstructions. Two patients had bilateral unicompartmental replacement (Table 1).

**Table 1:** Patient Demographics

Numbers (N)	7 uni compartmental, 2 Bi uni compartmental
Gender M:F	5:4

Age	56 (50 - 64)

Side Rt: Lt	2:7

Diagnosis	9 osteoarthritis with ACL deficiency

Compartment Replaced	Medial - 6, Lateral - 1

	Bilateral unicompartmental- 2

Prosthesis	Allegretto - 5

	Preservation -4

ACL Graft	BPT - 8, Hamstring - 1

### Clinical results

The average arc of flexion was 119° (range 85° to 135°) preoperatively and 125° (range 105° to 140°) at the time of final follow up.

All the clinical scores were found to improve post operatively (Table 2)

**Table 2:** Knee scores

	Pre Op	Follow up
WOMAC	45(35 - 52)	24 (21- 27)

Knee society Score	135 (64- 167)	196(190- 200)

Oxford knee score	23.5(20- 58)	11 (10- 12)

There were no signs of instability during the follow up of patients, with negative lachmann and pivot shift tests which were compared to the normal side. No patients in this group were reoperated.

### Radiographic results

The average preoperative deformity was 8° of varus (range, 3° of varus to 14° of varus) from the mechanical axis. The average postoperative alignment was 2° of varus (range, 2° of valgus to 10° of varus), for an average correction of 6°.

**Figure 1:**
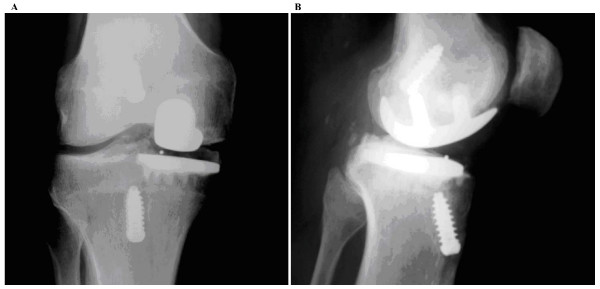
**Radiograph of unicompartmental replacement with ACL reconstruction**.

**Figure 2:**
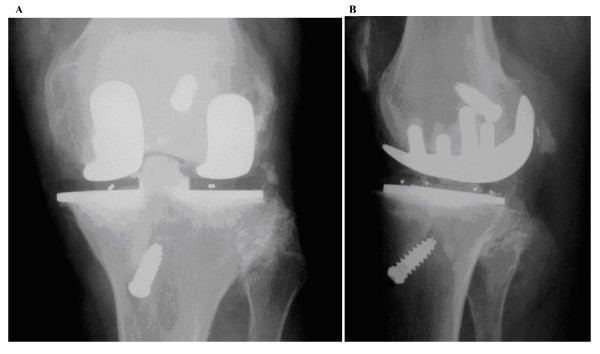
**Radiograph of bi unicompartmental replacement with ACL reconstruction**. A - Anteroposterior View, B - Lateral View.

At the time of final radiographic evaluation, no patient had evidence of component subsidence or pathological radiolucencies to suggest loosening. (Fig 1, 2).

## Discussion

The patient with an unstable knee and unicompartmental arthritis is a small but important subgroup. Resurfacing of the involved compartment alone may well relieve the pain but disabling instability would be likely to persist and the longevity of the implant may be compromised. Reconstruction of the ACL alone will often correct the instability but pain will persist.

The main concern about this combined procedure is log-term survival of the implant. A functional ACL is believed to play an integral role in the success of UKA [5,7,10], and [11] Goodfellow et al found that in their 103 unicompartmental cases; there were a significantly higher percentage of failures in knees with a deficient ACL (16.2%) than in knees with an intact ACL (4.8%). In a study of 301 meniscal arthroplasties, Goodfellow et al found a 6-year survival rate of 95% for knee with a normal ACL, whereas knees with a damaged or absent ACL demonstrated survival rate of only 81% [11].

Engh [16] reported that unicondylar arthroplasty may be an acceptable alterative for an inactive, elderly patient with an ACL deficient knee, but not for an active patient. As degenerative arthritis progresses in an ACL-deficient knee, adaptive changes alter the location of wear of the medial tibial plateau. The adaptive changes and altered kinematics that result from ACL deficiency probably are not altered after UKA. These adaptive changes limit subluxation and the giving way that occurs after a tear of the ACL. In a knee with a deficient ACL, articular surface wear characteristically involves the center or even posterior aspects of the medial tibial plateau. Such a wear pattern is indicative of the altered kinematics that results from the loss of ACL stability and compromised proprioception. Without these adaptive changes in active individuals, substantial loads occur across the knee with twisting and pivoting activities and may result in tibiofemoral subluxation.

The most common cause of failure of unicompartmental replacement was tibial component loosening [11]. Reconstruction of the ACL may prevent the failures associated with ACL deficiency [7].

It has also shown that normal kinematics is restored in the ACL deficient arthritic knee by combined ACLR and Oxford UKA. It is probably because the kinematics is restored that the patients who have had an ACLR and UKA have been able to achieve such a high level of function [17].

Tinius in his short term results of minimally invasive unicondylar knee arthroplasty with simultaneous ACL reconstruction in young patients had good outcome [18,19]. From our study the short term results of combined anterior cruciate ligament reconstruction and unicompartmental knee arthroplasty is technically feasible and provides good results in functionally unstable knees.

In our series we did not have any adverse radiological signs observed in relation to tibial component fixation. There is no pathological posterior femoral subluxation to cause eccentric loading and therefore loosening of the tibial component. If failure due to ligament instability can be avoided, the most important failure mechanism in this young and active patient group will be wear. There is a concerns that the excellent function may result in high physical demands on the knee, even when the patient is advised to restrict their activities [20]. It is therefore necessary to inform the patient of the risk of failure, even though revision to a TKR is relatively easy [21].

The short-term results of ACL reconstruction combined with unicompartmental knee arthroplasty in functionally unstable knees are good [22]. All patients have an excellent clinical outcome with resolution of both their arthritic pain and their functional instability.

## Conclusion

In this small series we have shown that instability can be corrected and pain relieved by this combined procedure.

It is too early to predict component loosening but at this stage we have not seen any evidence of early loosening as described by other authors performing unicompartmental arthroplasty in the unstable knee.

Therefore we believe that the combined approach is a viable option available for young active patients with symptomatic arthritis in whom the ACL deficiency is associated with functional instability.

At this early stage it should only be proceeded with after the patient fully understands the risks and benefits of the procedure and alterative treatment options.

## Competing interests

The authors declare that they have no competing interests.

## Authors' contributions

All authors had substantial contributions to conception and design of the study and giving final approval to the manuscript. SKRK, RR participated in the data acquisition and data interpretation and writing of the manuscript. All authors have read and approved the final manuscript.
